# Supplementary material for: Development and validation of a nomogram to predict postoperative pulmonary complications following thoracoscopic surgery
Source: PeerJ. 2021 Nov 4;9:e12366. doi: 10.7717/peerj.12366 (PMC8572520; doi:10.7717/peerj.12366)

Supplementary Table 1. The EPCO PPC definition used in this study

| Complication | Definition |
| --- | --- |
| Respiratory infection | The patient took antibiotics for suspected respiratory infection and met one or more of the following criteria: new or changed sputum, new or changed pulmonary turbidity, fever, white blood cell count > 12 × 10^9^/L. |
| Respiratory failure | PaO_2_ < 60 mmHg, PaO_2_:FiO_2_ ratio < 300 mmHg or arterial oxygen saturation (pulse oxygen saturation) < 90% after operation; oxygen therapy is needed. |
| Pleural effusion | Chest X-ray showed that the costoseptal angle became blunt, the contour of the diaphragm disappeared when the ipsilateral diaphragm was upright, the adjacent anatomical structure was displaced or the half chest (supine position) was blurred and turbid, and the vascular shadow remained. |
| Atelectasis | Pulmonary opacity: displacement of mediastinum, hilum or hemidiaphragm to the affected area, and compensatory hyperinflation of the adjacent non-atelectasis lung. |
| Pneumothorax | No blood vessel bed around the pleura. |
| Bronchospasm | Newly discovered expiratory wheezing treated with a bronchodilator. |
| Aspiration pneumonitis | Acute lung injury after inhalation of reflux gastric contents. |

Supplementary Table 2. PPCs details in this study.

| Complication | Number of cases |
| --- | --- |
| Total | 207 |
| Respiratory infection | 98 (0.473) |
| Respiratory failure | 8 (0.0386) |
| Pleural effusion | 154 (0.744) |
| Atelectasis | 12 (0.058) |
| Pneumothorax | 16 (0.077) |
| Bronchospasm | 0 |
| Aspiration pneumonitis | 0 |

Supplementary Figure 1. Flow chart of the study.


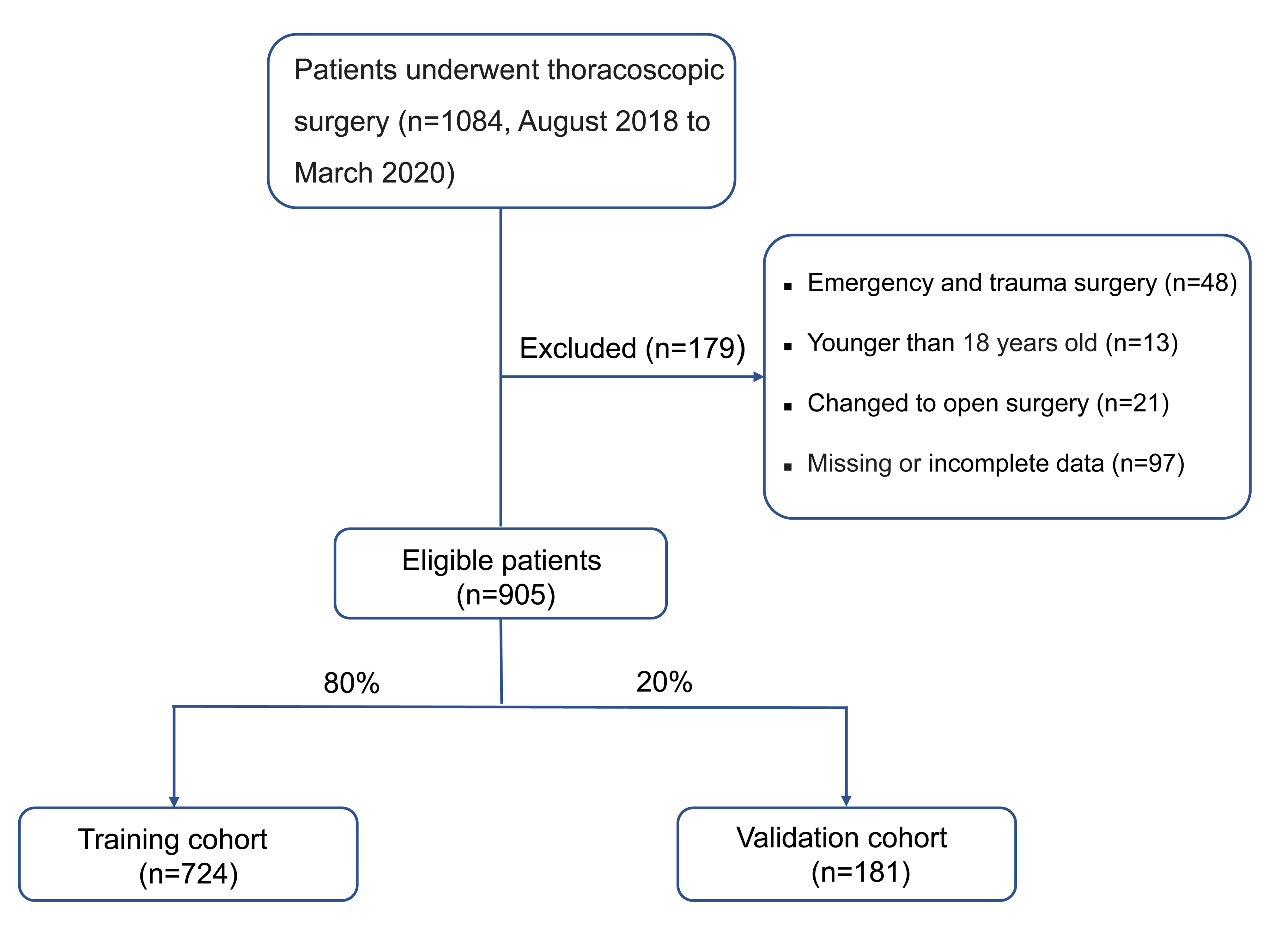

Supplement: Supplemental Information 3 [file peerj-09-12366-s003.docx]
