# Supplementary material for: Development and validation of a nomogram to predict postoperative pulmonary complications following thoracoscopic surgery
Source: PeerJ. 2021 Nov 4;9:e12366. doi: 10.7717/peerj.12366 (PMC8572520; doi:10.7717/peerj.12366)
Supplement: Supplemental Information 5 [file peerj-09-12366-s005.docx]

|  | **Section & Topic** | **Item** | **Development or Validation** | **Checklist Item** | **Reported on page #** |
| --- | --- | --- | --- | --- | --- |
|  |  |  |  |  |  |
|  | **Title and abstract** |  |  |  |  |
|  | **Title** | **1** | D;V | Identify the study as developing and/or validating a multivariable prediction model, the target population, and the outcome to be predicted | Page 1 |
|  | **Abstract** | **2** | D;V | Provide a summary of objectives, study design, setting, participants, sample size, predictors, outcome, statistical analysis, results, and conclusions. | Page 2-3 |
|  | **Introduction** |  |  |  |  |
|  | **Background and objectives** | **3a** | D;V | Explain the medical context (including whether diagnostic or prognostic) and rationale for developing or validating the multivariable prediction model, including references to existing models. | Page 4 |
|  |  | **3b** | D;V | Study objectives and hypotheses | Page 5 |
|  | **Methods** |  |  |  |  |
|  | **Source of data** | **4a** | D;V | Describe the study design or source of data (eg, randomized trial, cohort, or registry data), separately for the development and validation data sets, if applicable. | Page 5-6 |
|  |  | **4b** | D;V | Specify the key study dates, including start of accrual; end of accrual; and, if applicable, end of follow-up. | Page 5 |
|  | **Participants** | **5a** | D;V | Specify key elements of the study setting (eg, primary care, secondary care, general population) including number and location of centres. | Page 6 |
|  |  | **5b** | D;V | Describe eligibility criteria for participants. | Page 5-6 |
|  |  | **5c** | D;V | Give details of treatments received, if relevant. | Page 5-7 |
|  | **Outcome** | **6a** | D;V | Clearly define the outcome that is predicted by the prediction model, including how and when assessed. | Page 6-7 |
|  | **Predictors** | **7a** | D;V | Clearly define all predictors used in developing the multivariable prediction model, including how and when they were measured. | Page 6 |
|  |  | **7b** | D;V | Report any actions to blind assessment of predictors for the outcome and other predictors. | Page 6 |
|  | **Sample size** | **8** | D;V | Explain how the study size was arrived at. | Page 5 |
|  | **Missing data** | **9** | D;V | Describe how missing data were handled (eg, complete-case analysis, single imputation, multiple imputation) with details of any imputation method. | Page 7 |
|  | **Statistical analysis methods** | **10a** | D | Describe how predictors were handled in the analyses. | Page 7 |
|  |  | **10b** | D | Specify type of model, all model-building procedures (including any predictor selection), and method for internal validation. | Page 6-8 |
|  |  | **10c** | V | For validation, describe how the predictions were calculated. | Page 6-8 |
|  |  | **10d** | D;V | Specify all measures used to assess model performance and, if relevant, to compare multiple models. | Page 6-8 |
|  |  | **10e** | V | Describe any model updating (eg, recalibration) arising from the validation, if done. | Page 6-8 |
|  | **Risk groups** | **11** | D;V | Provide details on how risk groups were created, if done. | Page 6-8 |
|  | **Development vs. validation** | **12** | V | For validation, identify any differences from the development data in setting, eligibility criteria, outcome, and predictors. | Page 6-8 |
|  | **Results** |  |  |  |  |
|  | **Participants** | **13a** | D;V | Describe the ﬂow of participants through the study, including the number of participants with and without the outcome and, if applicable, a summary of the follow-up time. A diagram may be helpful. | Page 8-9 |
|  |  | **13b** | D;V | Describe the characteristics of the participants (basic demographics, clinical features, available predictors), including the number of participants with missing data for predictors and outcome. | Page 8-9 |
|  |  | **13c** | V | For validation, show a comparison with the development data of the distribution of important variables (demographics, predictors and outcome). | Page 8-10 |
|  |  | **14a** | D | Specify the number of participants and outcome events in each analysis. | Page 8-10 |
|  |  | **14b** | D | If done, report the unadjusted association between each candidate predictor and outcome. | Page 8-10 |
|  | **Model specifcation** | **15a** | D | Present the full prediction model to allow predictions for individuals (ie, all regression coeffcients, and model intercept or baseline survival at a given time point). | Page 8-10 |
|  |  | **15b** | D | Explain how to use the prediction model. | Page 10 |
|  | **Model performance** | **16** | D;V | Report performance measures (with CIs) for the prediction model. | Page 10 |
|  | **Model updating** | **17** | V | If done, report the results from any model updating (ie, model specifcation, model performance). | NA |
|  | **Discussion** |  |  |  |  |
|  | **Limitations** | **18** | D;V | Discuss any limitations of the study (such as nonrepresentative sample, few events per predictor, missing data). | Page 13 |
|  | **Interpretation** | **19a** | V | For validation, discuss the results with reference to performance in the development data, and any other validation data. | Page 11-13 |
|  |  | **19b** | D;V | Give an overall interpretation of the results, considering objectives, limitations,results from similar studies, and other relevant evidence. | Page 11-13 |
|  | **Implications** | **20** | D;V | Discuss the potential clinical use of the model and implications for future research. | Page 12-13 |
|  | **Other information** |  |  |  |  |
|  | **Supplementary information** | **21** | D;V | Provide information about the availability of supplementary resources, such as study protocol, Web calculator, and data sets. | Page 14 |
|  | **Funding** | **22** | D;V | Give the source of funding and the role of the funders for the present study. | Page 14 |
|  |  |  |  |  |  |

*Items relevant only to the development of a prediction model are denoted by D, items relating solely to a validation of a prediction model are denoted by V, and items relating to both are denoted D;V. We recommend using the TRIPOD Checklist in conjunction with the TRIPOD explanation and elaboration document.
